# Enhancement of reactive oxygen species production by ultra-short electron pulses

**DOI:** 10.1098/rsos.240898

**Published:** 2024-11-13

**Authors:** J. Tye, O. Solgaard, R. J. England, J. V. Trapp, A. Fielding, C. P. Brown

**Affiliations:** ^1^ MMPE, Faculty of Engineering, Queensland University of Technology, Brisbane, Queensland, Australia; ^2^ Centre for Biomedical Technology, Queensland University of Technology, Brisbane, Queensland, Australia; ^3^ Edward L. Ginzton Laboratory, Department of Electrical Engineering, Stanford University, Stanford, CA, USA; ^4^ SLAC National Accelerator Laboratory, Menlo Park, CA, USA; ^5^ School of Chemistry and Physics, Faculty of Science, Queensland University of Technology, Brisbane, Queensland, Australia; ^6^ Medical Engineering Research Facility, Queensland University of Technology, Brisbane, Queensland, Australia

**Keywords:** dielectric laser accelerator, electron-tissue interaction, electron radiotherapy, reactive oxygen species, pulse duration

## Abstract

The development of laser-driven accelerators-on-chip has provided an opportunity to miniaturize devices for electron radiotherapy delivery. Laser-driven accelerators produce highly time-compressed electron pulses, on the 100 fs to 1 ps scale. This delivers electrons at high peak power yet low average beam current compared with conventional delivery devices, which generate pulses of approximately 3 µs. The biophysical effects of this time structure, however, are unclear. Here, we use a Monte Carlo simulation approach to explore the effects of the electron beam time structure on the production of reactive oxygen species (ROS) in water. Our results show a power law increase in the generation of hydroxyl ions per deposited electron with decreasing pulse length over the pulse length range of 10 µs to 100 fs. Similar trends were observed for hydrogen peroxide, superoxide, hydroperoxyl, hydronium and solvated electrons. In practical terms, this indicates a fourfold increase in the efficiency of free radical production for sub-picosecond pulses, relative to that of conventional microsecond pulses, for the same number of deposited electrons.

## 1. Introduction

Surgical and radiation treatments for cancer can be complicated by proximity to sensitive tissues, degree of differentiation, rarity, level of invasiveness, difficulty in access or a challenging environment caused by previous treatment. Such challenges are often linked to poor prognosis [1], leading to a need for increased precision in treatment [2]. A prominent example is glioblastoma, which is characterized by extreme difficulty in access, surrounding tissue sensitivity [2] and a 7% survival rate at 5 years [1]. Similar challenges exist for advanced colorectal, prostate, synchronous and metachronous multiple primary tumours, with nerve sparing remaining a concern [3].

Advancing technological platforms have provided a basis to improve treatment precision and specificity. Improved precision through the dexterity, scaling of motion, vibration minimization and systems integration offered by surgical robots [4,5] has enabled improvements in patient outcomes across a range of oncological interventions [5]. Furthermore, accelerators delivering increased dose rates have demonstrated reduced radiation toxicity in healthy cells while maintaining, or potentially improving efficacy against tumours [6]. This ‘FLASH’ radiation approach involves dose rates at or above 40 Gy s^−1^, compared with conventional rates of the order of 10^−3^ to 10^−2^ Gy s^−1^ [6], potentially allowing the delivery of single-shot, high-dose fractions limiting radiation resistance and improving specificity in targeting tumours [6]. Despite promising *in vitro* results, technological advances are needed to enable the effective clinical application of these approaches, particularly for deep tissue.

**Figure 1.**
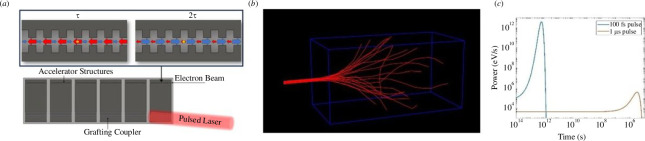
(*a*) Schematic of on-chip electron accelerator waveguide coupled with femtosecond pulsed laser. (*b*) Simulation of a 20-electron pulse using the TOPAS software, projecting an electron beam typical of an on-chip accelerator. (*c*) Log–log comparison of the power disparity.

Recent developments in accelerator technology offer new opportunities to extend this dose rate effect, tuning the energy exchange during radiation dosing and enabling dose delivery to deep or complex tumour sites. In particular, dielectric laser accelerators (DLAs) have demonstrated capability in generating attosecond pulse trains of electrons within the time envelope of the driving laser pulse, typically in the femtosecond range [7–12]. These accelerators use laser light, shaped by a dielectric waveguide, to create a near field that can accelerate charged particles such as electrons (figure 1) [7]. Furthermore, GeV m^−1^ acceleration gradients [8,9], on-chip manufacture, and coupling with miniaturized photocathodes [10] provide a potential to mount accelerators on robotic platforms to deliver the radiation beam to a tumour site with high precision, thus overcoming the scattering and penetration limitations of electron radiotherapy.

The inherent pulse characteristics from DLAs prompt the question of how the time structure of electron pulses, particularly in the ultra-fast (sub-picosecond) regime, affects the biochemical and biological response. Here, we examine the generation of ROS as a simulated function of the electron pulse time structure over the range of DLA, FLASH and conventional radiotherapy parameter space.

## 2. Method

Simulations were performed using the TOPAS-nBio v. 2.0 Monte Carlo package to trace particle trajectories, tracking energy exchange and free radical production from the physical electron interactions with the constituent atoms of the medium [13]. This implementation of the independent reaction times (IRT) Monte Carlo technique allows for calculation of time-dependent radiolytic yields and tracking of ROS via independent pair approximation to simulate reaction times between ROS created at the end of the pre-chemical stage. Prior to the main simulation run, the set-up was verified using matched molecules, reactions, reaction rate coefficients and diffusion coefficients from previous work [14].

The single pulse analysis within TOPAS-nBio was adapted to accumulate data from all particles within the pulse, allowing direct comparison. In response to the significant computational demands of this particular study, multi-threading was integrated to effectively reduce processing time. Furthermore, modifications were made to the original FLASH scorer [14] to facilitate inspection of the single pulse results. Global variables were implemented to preserve key outputs across the parallel threads. Following calculation of G values that represents the production of a specific ROS of interest by every 100 eV deposited into the target for each thread, a Mutex lock and counter were activated to ensure the sequential execution of concurrent processes or threads. This approach facilitated the aggregation of individual G values generated by each thread that could be managed and fed back into the single-thread design of the main code. The original conditional statement governing the processing of energy deposition and G values was revised, removing the dose parameter with the current count of histories scored, incremented by 1 to account for the initial count at 0. This value was assigned to a variable referred to as the ‘final count’, representing the total number of events to be processed divided by the number of active worker threads. This adjustment enabled the functioning of the code across both single-thread and multi-thread operations, ensuring the comprehensive scoring of all particles before initiating the final output calculations. The modified section of code is provided in electronic supplementary material.

All simulations used a 5 µm (full width at half maximum) Gaussian electron beam aligned with the z-axis into a 200 × 200 × 400 µm (x, y, z) pure water phantom, via a 100 µm air layer. Simulations were performed for a range of pulse durations (10^−13^–10^−6^ s), electron energies (10^4^–10^6^ eV) and number of electrons per pulse (1–100). Simulations allowed inter-track interaction and radical–radical recombination in the phantom. TOPAS output was analysed using in-house MATLAB code enabling a description of overall ROS production from each parameter set.

## 3. Results and discussion

Simulation results are presented for the generation of hydroxyl ions, responsible for 60–70% of cellular damage caused by ionizing radiation [15], from a narrow (Ø 5 mm) beam indicative of a diverged DLA output (figure 1*b*). Hydroxyl yields are normalized to the number of electrons contained in the individual pulse. Results for other ROS (hydrogen peroxide, superoxide, hydroperoxyl, hydronium), hydrogen and solvated electrons are provided in electronic supplementary material.

**Figure 2.**
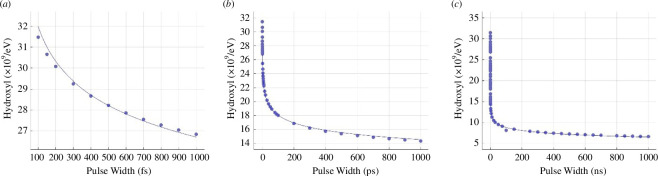
Hydroxyl production per 100 eV by a 100 keV, 10 electron single pulse with the changing of pulse duration ranging from 100 fs to 1 µs. Total hydroxyl production is plotted for pulse widths in the (*a*) femtosecond, (*b*) picosecond and (*c*) microsecond timescales. Data are plotted as points, with the power law fit plotted as a continuous line.

Hydroxyl production from a single pulse in water (figure 2) followed a power law trend ($7.0144\times 10^{7}\;\tau^{-0.05935}-9.4598\times 10^{7},$ $R^{2}=0.9994$, where τ is pulse width in seconds), increasing with decreasing pulse duration and rising steeply below approximately 50 ps. The few picoseconds to microseconds pulse width region corresponds to the timescale of the chemical stage of radiolysis [16], during which the ROS created by the electron track reacts and diffuses into the solution. The diffusion of the free radicals leads to the expansion of the track as the radicals react with surrounding molecules and with each other [16].

For pulse widths below the picosecond regime, corresponding to the physicochemical stage of radiolysis [16], efficiency was observed to increase at a rate slightly below that of the power law fit (figure 2*a*). The effect of this time structure was observed to be independent of both the number of electrons per pulse (electronic supplementary material, figure S1) and electron energy (electronic supplementary material, figure S2), with the fourfold increase in hydroxyl production per electron observed for picosecond versus microsecond pulse durations across the parameter space tested. Similar power-law trends were observed for hydrogen, solvated electrons and other ROS (electronic supplementary material, figures S3–S7), though a more linear increase was observed in superoxide and hydroperoxyl (electronic supplementary material, figures S8 and S9).

Together, these results suggest that the confinement of the electron beam in both space and time has a substantial effect on the dynamics of ROS production. Pulses in the hundreds of femtosecond regime typical of the thulium and holmium doped lasers used with DLAs, increase peak power by approximately 10^7^ compared with that of a conventional microsecond pulse. Here, the total energy from the electrons is deposited during the physicochemical to early chemical stages of radiolysis, ahead of the diffusion time. This acts to maintain the concentration of ROS during production, increasing probability of interaction between ROS. Such interactions may be observed in the yield of hydrogen peroxide (electronic supplementary material, figure S5), which is produced through radical–radical recombination. Confinement in time may further enable inter-track interactions within the pulse as a potential source of nonlinearity. In the microsecond regime, corresponding to the late chemical stages of radiolysis, the spread of energy deposition allows track expansion and the diffusion of ROS within the pulse duration, thus decreasing concentration and the probability of interaction. It is interesting to note that, within the parameter space tested here, increasing concentration via the number of electrons deposited did not give rise to the nonlinearities observed through concentration in time, instead showing the expected linear increase in ROS number of electrons. This indicates peak power, rather than the energy deposited from that peak, dominates ROS production.

The increase in efficiency of free radical production observed in this study for decreased pulse duration may provide mechanistic insight into experimental findings using shortened pulses. *In vitro* experiments have demonstrated increased rates of DNA damage and cell death under ultra-short electron pulses compared with conventional radiation [17]. Similar increases in the rate of DNA damage under time-compressed pulses have been observed in rats, together with a modified inflammatory response [18]. These cellular responses are also characteristic of FLASH doses [19], suggesting increased rates of free radical production as an alternative, yet related, hypothesis to that of oxygen tension in the FLASH effect [6]. Further investigation is needed to substantiate this proposition.

## 4. Conclusion

Our results may further inform future development in clinical accelerators to deliver radiation pulses below 50 ps width to enable more effective and efficient delivery of radiation. Specific to DLA development for oncology, the findings suggest electron energy and beam current as key limitations where, despite relatively high repetition rates, current outputs may need to increase for effective clinical application. Overall, we propose that the delivery of electrons within time-compressed pulses, particularly in the hundreds of femtoseconds to picosecond regime, will enable radiation doses to be substantially reduced for a given biological effect.

## Data Availability

The data and code that supports the findings of this study is available in Dryad [20]. Supplementary material is available online [21].
